# Immunomodulatory strategies for ex vivo immune cell expansion in adoptive cell therapy: translational and regulatory considerations for clinical manufacturing

**DOI:** 10.3389/fimmu.2026.1861621

**Published:** 2026-10-05

**Authors:** Weng Wan Chan, Estella Zhi Yu Lim, Deepak Choudhury

**Affiliations:** 1 Bioengineering and Automation, Bioprocessing Technology Institute (BTI), Agency for Science, Technology and Research (A*STAR), Singapore, Singapore; 2 Department of Food Science and Technology, National University of Singapore, Singapore, Singapore

**Keywords:** adoptive cell therapy (ACT), CAR-T cell manufacturing, ex vivo expansion, immunomodulation, NK cell manufacturing, regulatory frameworks

## Abstract

Ex vivo expansion is a fundamental yet challenging step in the manufacturing of adoptive cell therapies (ACT), as therapeutic success relies on generating enough functional immune cells while maintaining their phenotype, metabolic fitness, and persistence. This review provides a comprehensive overview of immunomodulatory strategies designed to enhance cell yield during ex vivo expansion, categorizing them into cell-intrinsic and cell-extrinsic approaches. Cell-intrinsic strategies, including gene editing and gene transfer technologies, enable precise modulation of survival pathways, proliferation, metabolic state, and resistance to exhaustion. These tools support the production of more potent and durable immune cell products. In contrast, cell-extrinsic strategies shape immune cell behavior through cytokine supplementation, checkpoint blockade, nutrient and metabolic modulation, feeder cell systems, synthetic activation platforms, and biophysical cues such as oxygen tension and bioreactor-controlled environments. Together, these strategies provide coordinated signals that direct activation, differentiation, and persistence during expansion in culture. The review also highlights key regulatory considerations, including distinctions between genetically and non-genetically modified products, risk assessments associated with human and animal-derived materials, ancillary material qualification, and process scalability for clinical translation. By integrating biological, engineering, and regulatory perspectives, this paper outlines principles for scalable immunomodulation platforms designed with regulatory considerations in mind to advance next-generation ACT manufacturing.

## 1 Introduction

### 1.1 What is cancer immunotherapy?

According to the World Health Organization (WHO), cancer is the leading cause of death globally, with an estimate of 10 million deaths in 2024, or nearly one in six deaths (1). The high mortality and morbidity associated with cancer underscore the critical need for more effective and targeted therapeutic approaches. One promising approach in recent years is cancer immunotherapy, which harnesses and enhances the body’s own immune system to identify and eliminate cancer cells (2). The advances in the understanding of immune mechanisms have driven the development of innovative therapies such as adoptive cell therapy (ACT), which uses the patient’s own immune cells to eliminate cancer (3–5). This approach may provide a more targeted and durable therapeutic response than some conventional treatments, although ACT has distinct toxicity profiles and can cause serious treatment-related adverse effects (6, 7).

Among the diverse approaches in cancer immunotherapy, this article focuses on adoptive cell therapy (ACT) (4).

### 1.2 What is adoptive cell therapy?

Adoptive Cell Therapy (ACT) relies on the natural role of the immune system to detect and destroy abnormal or infected cells, including cancer cells (8). However, in many cancer patients, the immune response is insufficient or suppressed by the tumor microenvironment. ACT seeks to overcome these limitations by isolating, modifying, and expanding immune cells outside the body before reinfusing them into the patient to amplify their anti-tumor activity (5).

While there are several distinct modalities within ACT, it can be broadly categorized into two main modalities: non-genetically modified ACT (such as Tumor-infiltrating Lymphocytes (TILs), Cytokine-induced killer (CIK), NK cell therapy, and NK92 cell therapy) (8–12) and genetically modified ACT (e.g. CAR-T, TCR-T, CAR-NK, CAR-NK92) (8, 13).

Non-genetically modified ACT involves immune cell isolation, activation, and expansion without genetic alteration. Genetically modified ACT adds an ex vivo genetic modification step typically before expansion to enhance specificity, persistence, or effector function.

Despite its clinical promise, ACT faces significant manufacturing challenges. Therapeutic efficacy is highly dependent on the ability to expand immune cells ex vivo to clinically relevant doses while preserving cell identity, functionality, and safety. Additionally, ex vivo expansion can induce immune cell overstimulation and exhaustion, leading to reduced function and proliferative capacity (14–16).

At its core, ACT manufacturing involves sourcing immune cells, their ex vivo activation or genetic modification and expansion, and subsequent administration to the patient to enhance anti-tumor activity. While significant attention has been given to the biological mechanisms governing immune cell activation and persistence (8), successful translation of adoptive cell therapies requires consideration of both manufacturing feasibility and regulatory compliance (17–19). The choice of immunomodulatory strategy impacts not only cell expansion and functionality, but also product characterization, process control, scalability, and regulatory oversight. Therefore, understanding the biological, engineering, and regulatory implications of these approaches is essential for the clinical implementation and commercialization of ACT products. This review aims to provide an overview of immunomodulatory techniques used during ex vivo expansion in ACT manufacturing with a focus on strategies for improving cell yield. We categorize these techniques into two major classes: cell-intrinsic strategies and cell-extrinsic strategies. In addition to summarizing their biological mechanisms and applications, we discuss the manufacturing and regulatory considerations associated with their implementation in ACT production. By outlining the current landscape of immunomodulatory techniques with manufacturing and regulatory considerations, this review aims to guide future innovation in ACT manufacturing and support the development of safer and more effective cellular therapies.

An overview of the immunomodulatory techniques in ACT is illustrated in **Figure 1**.

**Figure 1:**
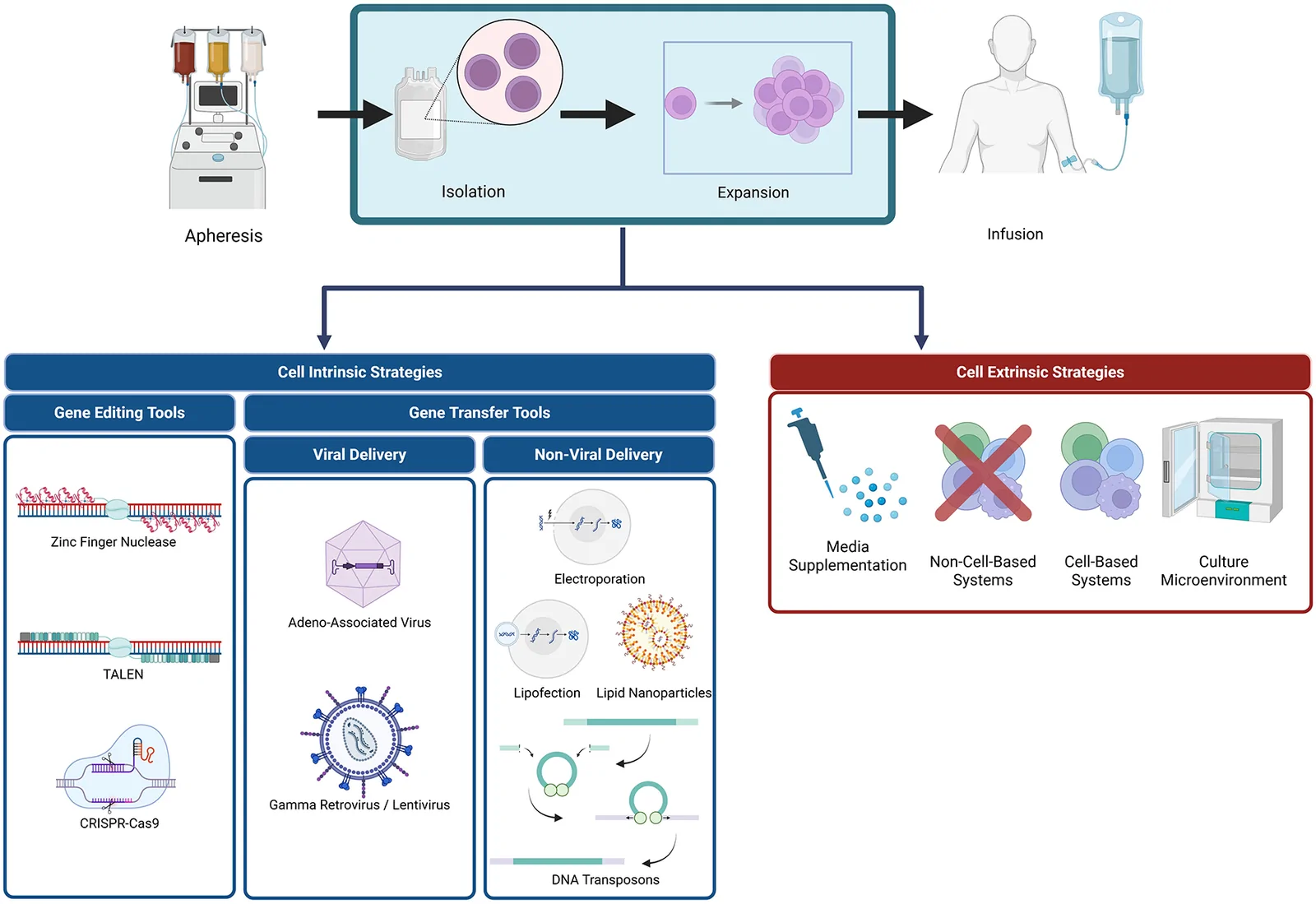
Overview of the immunomodulatory techniques in ACT. Created in BioRender. Choudhury, D. (2026) https://BioRender.com/24qvllc.

## 2 Immunomodulatory strategies for ex vivo expansion

The ex vivo expansion step aims to generate sufficient cell quantities with modifiable functions and durable therapeutic response (17). This is achieved by adopting immunomodulatory strategies to manipulate the immune cells ex vivo. These strategies enhance the activation and proliferation of therapeutic cells prior to reinfusion leading to improved efficacy (20). These strategies can be broadly categorized into cell-intrinsic strategies (e.g. genetic modifications that reprogram cellular responses) and cell-extrinsic strategies (e.g. media supplementation with cytokines, cell-based/non-cell-based activation systems, and biophysical cues that influence cell behavior).

### 2.1 Cell-intrinsic strategies

Prolonged stimulation in the process of ex vivo expansion of immune cells often leads to activation-induced exhaustion, metabolic decline, and apoptosis (21). These effects limit both cell yield and functional quality. Cell-intrinsic strategies in immune cell ex vivo expansion involve genetic engineering that reprograms intracellular signaling pathways and cellular behavior. These approaches aim to improve cell survival, proliferation, and metabolic fitness (22). From a manufacturing and regulatory perspective, cell-intrinsic strategies are generally associated with a higher degree of product manipulation because they involve direct genetic modification of therapeutic cells (19, 23, 24). While these approaches offer precise control over cellular phenotype and function, they also introduce additional requirements for product characterization, process control, and safety assessment. Depending on the modification strategy employed, manufacturers may be required to evaluate parameters such as editing efficiency, vector copy number, genomic stability, off-target effects, and long-term persistence.

The modification of immune cell biology is achieved via gene editing and gene transfer technologies. Gene editing technologies define the desired genomic modification, whereas gene transfer platforms deliver either the editing machinery or donor templates required to implement those changes (22, 25). While gene editing tools and gene transfer tools are presented here as distinct conceptual categories, they are frequently used in combination during ACT manufacturing. The selection of these tools must balance biological performance with manufacturing complexity, scalability, and regulatory expectations for clinical translation. The following sections highlight the common gene editing and transfer tools in ACT.

#### 2.1.1 Gene editing tools

Gene editing allows for precise and programmable modifications of immune cell genomes, enabling researchers to enhance proliferation, persistence, and functional performance during ex vivo expansion (26). This approach typically relies on programmable nucleases, such as zinc finger nucleases (ZFNs), Transcription activator-like effector nucleases (TALENs), or Clustered Regularly Interspaced Short Palindromic Repeats (CRISPR) and CRISPR-associated protein 9 (CRISPR-Cas9) which introduce double-strand breaks at specific genomic loci (26). The cell’s endogenous repair machinery then processes these breaks, resulting in either gene disruption (knockout) or targeted gene insertion (knock-in) (27, 28).

ZFNs were used to target the TCR β and α chain gene to disrupt the expression of CD3-TCR on T cells via adenoviral vectors. These modified T cells were then transduced by lentiviral vectors to express tumor specific TCR (29). This approach reduced the issues of GvHD and provided the means for selection and expansion of an enriched population of antigen-specific T cells.

TALENs were used to target the TRAC locus via mRNA electroporation (30). This resulted in the disruption of the TCRα chain and in turn the cells did not express cell surface CD3/TCR complexes. Specifically, TRAC disruption was followed by expression of anti-CD3ϵ CAR (3CAR) via lentiviral transduction. This approach was used to produce 3CAR T-cells while avoiding T-on-T killing and fratricide effects. The cells cultured this way proliferated by 100-fold over the span of 11 days using G-Rex flasks, allowing large-scale production of effector cells (30).

CRISPR-Cas9 was used in the deletion of the CISH gene in iPSC-derived NK cells (31). The CISH knock out iPSC-NK cells resulted in better expansion and cytotoxicity when compared to unmodified iPSC-NK cells under cytokine limiting conditions.

A summary table of the common gene-editing tools is highlighted in **Table 1**.

**Table 1:** Comparison of genome-editing platforms: ZFN, TALEN, and CRISPR-Cas9.

	Zinc finger nucleases (ZFN)	Transcription activator-like effector nucleases (TALEN)	CRISPR-Cas9	Ref.
Type	Protein-based nuclease system	Protein-based nuclease system	RNA-protein based system	(32)
Structure and Mechanism	Paired zinc-finger DNA-binding domains fused to the FokI endonuclease, where each finger recognizes 3–4 bp. Binding of ZFN pairs on opposite DNA strands enables FokI dimerization, generating a double-strand break (DSB) with 5′ overhangs that is repaired by NHEJ or HDR.	Paired transcription activator-like effector (TALE) repeat arrays fused to the FokI endonuclease, with each repeat conferring single-base specificity. Binding to opposing target sites with a 12–30 bp spacer allows FokI dimerization and formation of a DSB with 5′ overhangs, repaired via NHEJ or HDR.	An RNA-guided Cas9 endonuclease directed by a single-guide RNA (sgRNA) containing a 20-nt target-complementary sequence. Cas9 introduces a PAM-dependent DSB approximately three base pairs upstream of the PAM, followed by repair through NHEJ or HDR.	(32–36)
Specificity (Target Sequence)	Higher 18–36 bp per ZFN pair	Highest 28–40 bp per TALEN pair	High 20bp guide sequence	(25, 33, 35, 37)
Efficiency	Normal	Normal	High Allow for multiplexing	(32, 38, 39)
Engineering Feasibility	Low ZFNs require custom protein engineering for each target sequence. Target selection depends on context-specific DNA–protein interactions and often requires extensive optimization. This results in long design–build cycles	High TALE design follows repeat-variable diresidue (RVD)–base pairing rules, enabling predictable DNA recognition. However, each new target still requires construction of a new protein pair, limiting throughput.	Highest Targeting specificity is determined by crRNA or gRNA sequence design rather than protein engineering. Guides can be rapidly designed, synthesized, and iterated, enabling high scalability and multiplexed genome editing.	(32, 34, 39–41)
Typical Development time	Longest (~7 to 15 days)	Long (~5 to 7 days)	Short (~1 to 3 days)	(42)
Cost	Highest	High	Low	(34, 38, 41)
Ease of Delivery	Highest: ZFN expression elementss are small (~1 kb). This makes packaging and delivery straightforward.	Low: The cDNA encoding TALEN is large (~3 kb), compared with ZFNs (~1 kb). Its size makes delivery and expression more challenging. Viral vectors are especially constrained by cargo size limits.	High: SpCas9 is large and can cause packaging issues for viral delivery. However, smaller Cas9 orthologs are available. Multiple delivery strategies exist, including viral vectors and RNA or protein electroporation for Cas9/sgRNA components.	(25, 33–36)

ZFNs and TALENs require the development of target-specific DNA-binding proteins, increasing design complexity, development timelines and manufacturing costs; CRISPR-Cas9 utilizes programmable guide RNAs that enable rapid retargeting and efficient multiplex genome editing (32, 33, 38). Although ZFNs and TALENs have been associated with high specificity, advances in guide RNA design and high-fidelity Cas variants have substantially improved the precision of CRISPR-Cas9 (33, 37). For gene edited products, manufacturing and regulatory considerations are particularly important. All gene-editing platforms require comprehensive characterization to evaluate editing efficiency, specificity, genomic integrity, and product safety (23, 24). However, the multiplexing capability of CRISPR-Cas9 may introduce additional considerations, including the assessment of off-target events, large deletions, and chromosomal rearrangements. Therefore, selection of an editing platform should balance editing flexibility, manufacturability, and scalability with the regulatory and analytical requirements necessary to ensure product quality and safety.

As an example of gene-editing-specific regulatory considerations, UCART19, an allogeneic anti-CD19 CAR-T product, illustrates the type of preclinical assessment required when multiplex genome editing is used (43, 44). UCART19 incorporates TALEN-mediated disruption of endogenous loci, including TRAC and CD52. *In vitro* studies were conducted to evaluate the risk of off-target DNA cleavage and to characterize chromosomal translocations arising between the TRAC and CD52 loci following multiplex gene editing.

#### 2.1.2 Gene transfer tools

Gene transfer tools are fundamental to implementing cell-intrinsic engineering strategies during ex vivo expansion, as they enable the introduction of genetic cargo or gene-editing machinery into immune cells with the required efficiency, safety, and functional precision. The method chosen for gene delivery directly influences transduction efficiency, cell viability, expression kinetics, and ultimately the quality of the final therapeutic product. Broadly, gene transfer tools fall into two categories: viral vectors, which take advantage of virus’ innate ability to transduce cells, and non-viral delivery systems, which use both physical and chemical methods to deliver genetic material (45).

Viral vectors are commonly used to transduce the expression of CAR on the immune cells of interest during ex vivo expansion. Currently, the commonly used viral vectors to introduce CAR in the commercial approved CAR-T therapies are the gamma-retroviral vector and the lentiviral vectors (46). Both viral vectors can result in stable integration into host cell genome enabling stable and long-term expression of therapeutic transgenes such as CARs or TCRs. However, the same integration event that supports long-term expression also introduces regulatory concerns regarding insertional mutagenesis (23, 24). As a result, manufacturers must perform vector copy number analysis, vector characterization, and testing for replication-competent viruses while ensuring GMP-compliant vector production.

Gamma-retroviral vectors can only integrate into the host genome during cell division, whereas lentiviral vectors are able to transduce both proliferating and non-proliferating cells. While adeno-associated virus (AAV) vector is also used to transduce both dividing and non-dividing cells. AAVs are generally not used for standard CAR-T production due to their non-integrating nature, which leads to reduced CAR expression as T cells proliferate. However, AAVs are increasingly utilized to deliver donor templates for targeted CAR integration via CRISPR/Cas9 (47, 48) or transposon system (49).

Despite their efficiency, viral systems present notable challenges. Integration-dependent vectors carry a residual risk of insertional mutagenesis if integrated near oncogenes or tumor suppressor loci. Additionally, large-scale viral production is labor-intensive, costly, and requires strict GMP-compliant manufacturing workflows limiting scalability.

Non-viral delivery methods can support both transient and stable genetic modification, additionally they have lower immunogenicity and minimal or absent risk of insertional mutagenesis (50).

Non-viral stable gene insertion can be achieved via DNA transposon systems (such as Sleeping Beauty and piggyBac) through a “cut-and-paste” mechanism. A transposase enzyme recognizes terminal inverted repeats (TIRs), excises the transposon, and integrates it into the host genome. These systems enable stable gene insertion to introduce CAR for both NK cells and T cell as an alternative to viral vector transduction (51, 52).

The methods to deliver cargo (such as nucleic acid, proteins, small molecules) across the cell membrane can be divided into physical and chemical methods.

Physical methods use mechanical energy to disrupt the cell membrane to allow the genetic material to enter the cell whereas chemical methods rely on the molecular interactions between the membrane and the delivery vessel to bypass the cell membrane. While non-viral methods have lower immunogenicity and production cost compared to viral vectors, their transfection efficiency is lower compared to transduction efficiency of viral vectors.

Electroporation is a physical method where a strong electric pulse is used to create temporary pores in the cell membrane to allow the desired cargo (e.g. nucleic acid, proteins, or drugs) to enter the cell. This method was used to produce high-affinity NK cells (haNK) which expressed a high affinity CD16 receptor as well as endoplasmic reticular IL-2 which allowed the cells to be expanded without IL-2 supplementation (53). A modified electroporation technique known as Nucleofection can deliver nucleic acid cargo directly into the nucleus. Using the nucleofection technique, non-viral delivery of Cas9 RNP and DNA template achieved robust triple knockout and knock-in of affinity tag (54).

Another physical method is particle-mediated gene transfer, in which particles coated with genetic material are bombarded onto cells. This approach was used to transfect NK92 cell to be IL-2 independent. Although stable genomic integration of the plasmid vector occurred only as a rare event, it resulted in the establishment of the NK-92MI and NK-92CI cell lines. These variant cell lines were able to expand without the need for exogenous supplement of IL-2 supporting the off-the-shelf NK92 cell therapy (55, 56).

Lipid based systems include lipofection systems and lipid nanoparticles (LNPs). Lipid based systems encapsulate mRNA within a lipid shell, which protects the cargo from degradation and facilitates cellular uptake via endocytosis. Following endocytosis, endosomal acidification triggers release of mRNA into the cytosol (57). mRNA-LNP transfection following a >8000 fold expansion of NK cells resulted in >75% CAR-positive NK cells and significantly decreased tumor growth in an *in vivo* mouse model (58).

Alternatively, polymer-based delivery systems can be used, in which cationic polymers interact with the negatively charged nucleic acids through electrostatic interactions to form nanoscale polyplexes. Similar to lipid-based systems, these nanoparticles protect the cargo from degradation and deliver it into the cell via endocytosis (59). In a study, cell-targeting mRNA polyplex nanocarriers were fabricated using polymers poly(β-amino ester) (PBAE) and CD3-antibody conjugated poly-glutamic acid (PGA). This enabled >80% transfection with transgene expression detectable as early as 5 hours post-transfection (60).

A summary of the tools used for gene delivery is provided in **Table 2**.

**Table 2:** Comparison of viral and non-viral systems used in gene transfer.

Type		Structure and mechanism	Advantages	Disadvantages	Typical cargo	Expression duration	Ref
Viral	Gamma-Retrovirus	Enveloped RNA virus that integrates into the host genome during cell division	Stable expression; efficient in rapidly dividing cells	Can only infect actively dividing cells. Insertional mutagenesis risk	~8–10 kb	Stable	(46, 57, 61, 62)
	Lentiviral Vector (LV)	LVs are a subclass of retroviruses capable of integrating their genetic material into the host genome in dividing and non-dividing cells	High efficiency in primary cells; stable expression; clinically established	Insertional mutagenesis risk	~8–10 kb	Stable	(46, 57, 61, 62)
	Adeno-associated virus (AAV)	Non-enveloped ssDNA virus that persists mainly as episomal concatemers capable of infecting both dividing and non-dividing cells	Does not integrate into the host genome, reducing the risk of insertional mutagenesis.	Limited cargo size	~5 kb	Transient in dividing cells Stable in non-dividing cells	(46, 57, 61–63)
Non – Viral	Electroporation	Electrical pulses transiently open pores in the cell membrane to deliver nucleic acids and proteins A modified electroporation technique known as nucleofection enables delivery of cargo directly into the nucleus of cells	Suitable for most cell types and allows for large cargo delivery. The ability to perform transient and gene insertions and support stable gene insertions with gene editing tools	Stressful process for cells, leading to a cell death.	DNA/RNA/Protein	Stable/ Transient	(64–66)
	Lipid Nanoparticles (LNPs)	Ionizable lipids encapsulate nucleic acids for endocytosis-mediated uptake	Efficient gene delivery and induce stable transfection with gene editing tools. Capable of activating and transfecting cell in a single step Tunable surface composition Low toxicity	Risk for excessive inflammatory response	DNA/RNA/Protein	Stable/ Transient	(65–67)
	Lipofection	Cationic lipid–nucleic acid complexes enter cells via membrane fusion or endocytosis	Low cost Simple	Higher toxicity than LNPs Relatively low transfection rates.	DNA/RNA/proteins	Transient	(64, 66)
	DNA Transposons	DNA transposons, also known as transposable elements, are DNA fragments that move within the genome. The transposon and a transposase enzyme responsible for excision and reintegration are delivered into the cytosol. Commonly used systems for gene editing include the Sleeping Beauty (SB) and piggyBac (PB) transposons, both of which enable stable genomic integration.	Large cargo capacity Stable expression	Insertional mutagenesis risk, Requires Transposase delivery	DNA (~10kb – 100kb)	Stable	(68, 69)

The choice of gene-transfer technology has important implications for manufacturing scalability, product consistency, and regulatory compliance. Viral vectors remain the industry standard due to their transduction efficiency (46, 61). However, their use is associated with complex GMP-compliant manufacturing processes, extensive vector characterization requirements, and rigorous testing to exclude replication-competent viruses. Integration-based systems such as lentiviral and γ-retroviral vectors additionally require assessments of insertional mutagenesis risk and vector copy number (23, 24). In contrast, non-viral gene delivery systems offer potential advantages in terms of reduced manufacturing costs, simplified scalability, and less complex supply chain management (64, 65). However, these approaches often require further optimization to achieve gene transfer efficiencies that are comparable to those of viral vectors and sufficient for clinical applications (50, 65). The regulatory considerations for the gene transfer tools include the assessment of process robustness, reproducibility, and the ability to consistently meet predefined critical quality attributes in addition to the biological efficacy of the ACT product (19, 23, 24).

The same UCART19 product also provides an example of gene-transfer-specific considerations, as CAR expression was introduced using a lentiviral vector (43, 44). Pre-clinical studies both *in vitro* and *in vivo* were conducted to demonstrate the potential safety profile of the product: Specificity of the 4g7 scFv component of UCART19 to its intended CD19 antigen (*in vitro*), as well as risk of Graft-versus-Host Disease (GvHD) (*in vivo*). The risk of tumorigenicity via the use of lentiviral vector was mitigated by checking for replication competent lentivirus absences in release criteria and using third generation vectors, where the lentiviral proteins are provided by helper plasmids. The risk of insertional mutagenesis is mitigated using a non-replicative self-inactivating (SIN) lentiviral delivery vector. Furthermore, following clinical trials, the patients are enrolled into Long-Term Follow-Up study for 15 years from time of last infusion in order to be monitored for their safety as part of regulatory requirements for gene therapy products (23, 24, 43, 70).

### 2.2 Cell-extrinsic strategies

While cell-intrinsic strategies directly reprogram the internal machinery of immune cells, cell-extrinsic approaches focus on directing cell fate by controlling the external environment in which cells are expanded. These interventions provide signals that influence activation, proliferation, and differentiation, ensuring the generation of a therapeutic product with optimal functionality and persistence. As immune cells are highly sensitive to their surrounding biochemical and biophysical context (70), extrinsic modulation plays a decisive role in steering cell fate during ex vivo culture. By fine-tuning cytokine exposure, cellular interactions, synthetic stimulation platforms, and the culture microenvironment, cell-extrinsic strategies can enhance the efficiency, consistency, and scalability of immune cell expansion processes.

Unlike the cell-intrinsic strategies, the main regulatory considerations for cell-extrinsic strategies generally relate to ancillary material qualification, contamination control, process reproducibility, scalability, and comparability during process changes (19, 71–74). Therefore, successful implementation of cell-extrinsic immunomodulation requires alignment of biological performance with validated manufacturing inputs and regulatory expectations for clinical translation.

#### 2.2.1 Media supplementation

Cytokines play a key role in immunomodulation. They are the key drivers of immune cell activation, growth, and differentiation (75). By controlling the cytokines introduced during ex vivo cell culture, the proliferation and expansion of a desired phenotype of immune cell for adoptive cell therapy can be achieved. Culture media supplemented with γ-chain cytokines, including IL-2, IL-7, IL-15 and IL-21, have been shown to promote the expansion of CAR-T cells with enhanced proliferative capacity, improved metabolic fitness, and reduced terminal differentiation (76). A relatively modest expansion of NK cells was achieved using a multicytokine combination IL-2, IL-15, and IL-21 in a feeder-free condition (77, 78).

Immune checkpoints are inhibitory immunoreceptors that regulate the degree of immune activation. The use of immune checkpoint inhibitors has also been used to support ex vivo expansion of immune cells. Exhaustion markers (such as PD-1, LAG3, CTLA4) are expressed in CD8+ effector T-cells upon chronic stimulation (79). It was shown that PD-1 blockade restored impaired function of ex vivo expanded CD8+ T cells and had increased cytotoxic activity against EpCAM+PD-L1+ tumor cells (80).

Nutrient and metabolic modulation is another strategy in ex vivo expansion of immune cells. Urolithin A (UA) was shown to be effective for expanding CAR-expressing T_SCM_ which are desired for expressing less exhausted cells within the tumor microenvironment (81). The study showed that UA drives T_SCM_ expansion by activating mitophagy-dependent Wnt/β-catenin signaling, thereby enhancing T-cell metabolic fitness and anti-tumor efficacy. Another study demonstrated that the glutamine antagonist 6-Diazo-5-oxo-l-norleucine (DON) shifts CAR-T cell metabolism by suppressing glycolysis and promoting fatty-acid–supported mitochondrial oxidation, resulting in enriched naïve and central memory T-cell subsets (82). In another study, CAR-NK92 cells were shown to have weaker proliferation and diminished redox-related amino acid metabolism compared to parental NK-92 cells. N-acetylcysteine (NAC) improved their intracellular redox balance by reducing reactive oxygen species (ROS) levels, and supplementation with 1 mM NAC significantly enhanced CAR-NK92 expansion ex vivo (13).

Cytokines, checkpoint inhibitors, and metabolic modulators used during ex vivo expansion are typically classified as ancillary materials and therefore require qualification for clinical manufacturing. Their source, purity, batch-to-batch consistency, and intended use can substantially influence both cell expansion and final product quality (72–74). The use of research-grade reagents may be acceptable during early development but often becomes problematic during later-stage clinical development and commercialization (72). Manufacturers must therefore establish strategies for transitioning to qualified GMP-grade reagents and demonstrate that changes in media formulations do not adversely affect critical quality attributes or product comparability (19). Regulatory agencies also expect mechanistic justification and risk assessments when introducing novel metabolic modulators or checkpoint-blocking agents into manufacturing workflows (19).

#### 2.2.2 Cell-based activation systems

Cell-based stimulation systems drive ex vivo immune cell expansion by recreating the complex, multi-signal environment required for lymphocyte activation and proliferation using feeder cells. By providing coordinated co-stimulatory cues and cytokine support, these systems promote robust proliferation and help to generate the desired immune cell phenotype (83).

In TIL therapy, tumor biopsy samples are harvested from the patient and the lymphocytes are isolated from these tumors and expanded using irradiated PBMC feeder cells pooled from healthy donors and cytokine stimulation (84). However, a large number of feeder cells are required (up to 10^10^ per patient treated), additionally the pooled PBMC from the multiple donor result in lot-to-lot heterogeneity resulting in inconsistent activation of TILs (85). To address these limitations, genetically engineered feeder cell lines have been developed.

The engineered feeder cells such as modified K562 can be engineered to express defined peptide-major histocompatibility complex (pMHC) molecules, co-stimulatory molecules like CD80, CD86, or 4-1BB ligand (4-1BBL), and membrane-bound cytokines including IL-15 or IL-21 (86, 87). In preclinical and clinical studies, K562-based feeder cells have enabled robust expansion of antigen-specific T cells, including NY-ESO-1-specific and MART-1-specific CD8+ T cells, generating cell products with high proliferative capacity (88). These engineered K562 cells have also been used in the expansion of NK cells where engineered-K562 feeder cells simultaneously co-expressed mbIL-21, mbIL-15 and 4-1BBL resulted in a 17902-fold increase in NK cell population from PBMCs within two weeks (89).

Feeder cell systems present unique regulatory challenges because they introduce additional biological materials into the manufacturing process (71). Human- or animal-derived feeder cells may increase the risk of adventitious agent transmission and therefore require extensive characterization, qualification, and traceability. Even engineered feeder cell lines capable of providing standardized activation signals require demonstration of identity, stability, and absence of replication-competent contaminants (71). Relevant quality-control parameters may include passage limits, sterility and mycoplasma testing, consistent expression of engineered stimulatory molecules, validated irradiation or inactivation, and functional potency in supporting immune-cell expansion. Furthermore, feeder cell removal strategies and residual feeder cell testing may be required before product release (19, 71).

Overall, cell-based stimulation systems play a central role in driving ex vivo immune cell expansion by integrating proliferative, survival, and differentiation cues that are difficult to replicate using soluble factors alone. While biological variability and manufacturing complexity limit the scalability of primary cell-based systems, engineered cell lines provide a more controllable approach to achieving high-yield, high-quality expansion. Though feeder cell systems can achieve exceptional expansion yields, the additional manufacturing controls and regulatory requirements associated with feeder cell materials can complicate process validation and scale-up efforts, particularly when compared with feeder-free alternatives (71).

#### 2.2.3 Non-cell-based activation systems

To overcome the variability, scalability constraints, and regulatory complexity associated with living feeder cells, non-cell-based stimulation systems have been developed to deliver defined, reproducible activation signals that drive ex vivo immune cell expansion. Unlike cell-based platforms, these systems provide control over the identity, density, and duration of stimulatory cues, enabling consistent proliferation across large-scale manufacturing workflows. Some of these systems include coated 2D culture surfaces (90), ligand coated scaffold/carriers (91–93), magnetic microbead with conjugated ligands (94, 95), and soluble activators (e.g. plasma membrane particles (96), engineered fusion proteins (97), antibody complexes (98), colloidal reagents (99)).

Antibody-coated 2D surfaces targeting NK-cell–activating receptors have been used to stimulate and restimulate NK cells before expansion in suspension cultures, achieving more than 1,000-fold expansion (90). In current commercial T-cell manufacturing, magnetic microbeads are widely used to provide activation signals and can be easily removed after expansion. Modulating the ratio of anti-CD3 to anti-CD28 antibodies adjusts the strength of stimulation; for example, a 2:1 CD3:CD28 ratio yields approximately 148-fold expansion by day 11 (94). Magnetic beads functionalized with anti-CD3/CD28 via polyisocyanopeptide immunobrushes further enhance proliferation while requiring fewer antibodies per bead (95). Aside from magnetic beads, ligand coatings have been applied to alternative carriers such as alginate hydrogels in the Cloudz Microsphere system, which enables removal of the carriers using a chelating agent after feeder-free NK cell expansion (91). Other carriers include lipid microbubbles and mesoporous silica microrods supporting lipid bilayers decorated with CD3/CD28 antibodies, both of which have been used to activate T cells prior to expansion (92, 93).

Soluble activators can also directly provide stimulatory signals. Plasma membrane particles derived from modified feeder cells (K562-41BBL-mbIL21) and engineered fusion proteins (HCW9101, HCW9206, HCW9107) have supported NK cell expansion by ~8,200-fold and ~4,000-fold over 14 days, respectively (96, 97). For T-cell activation, commercial solutions include TransAct™, a colloidal polymer matrix presenting anti-CD3/CD28 agonists, and ImmunoCult™, a CD3/CD28/CD2 antibody complex (98, 99).

Collectively, non-cell-based systems drive ex vivo immune cell expansion by delivering consistent and programmable activation signals that promote high proliferative output while enabling control over phenotypic quality. Compared with feeder cell approaches, non-cell-based activation systems generally offer superior reproducibility and regulatory tractability because the stimulatory components are more readily defined and characterized. Activation beads, synthetic scaffolds, antibody complexes, and engineered stimulatory particles can be produced using standardized manufacturing processes and are often easier to qualify as ancillary materials (73, 74). Nevertheless, manufacturers must demonstrate consistent activation performance, effective removal of process-related materials where applicable, and an absence of adverse effects on final product quality (100). The well-defined nature of these systems is particularly advantageous during process scale-up and comparability assessments, making them attractive options for commercial manufacturing (101, 102).

#### 2.2.4 Culture microenvironment

Beyond stimulatory signals, the biophysical properties of the culture environment play a critical role in shaping T cell expansion, differentiation, and functional persistence during ex vivo ACT protocols. Optimizing parameters such as oxygen tension, pH, and substrate stiffness can significantly improve the quality and durability of expanded immune cells.

Oxygen tension is a key factor influencing T cell metabolism and differentiation (103–105). While standard laboratory incubators operate at atmospheric oxygen levels (~20%, normoxia), most tissues in the body, including lymphoid organs, are physiologically hypoxic (1-5% O_2_) (106). Culturing T cells under hypoxic conditions can profoundly impact their phenotype (105). Hypoxic culture conditions have been shown to promote a less-differentiated, stem-like memory phenotype (T_SCM_), which is highly desirable for ACT as these cells possess superior capacity for self-renewal and long-term persistence after infusion (104, 106). Furthermore, mimicking the low-oxygen environment of solid tumors ex vivo can pre-condition T cells for better survival and function upon encountering the tumor microenvironment (107). However, the clinical translation of hypoxic culture requires specialized incubators or bioreactors capable of precise gas control, which adds cost and complexity to GMP manufacturing (108). The optimal oxygen concentration can vary depending on the cell type, and the desired balance between memory and effector phenotypes (103). A major challenge is to define the ideal oxygen level and duration of exposure that maximizes therapeutic benefits without compromising cell viability or inducing terminal exhaustion (104).

Bioreactor-based culture systems represent an integrated approach to microenvironment optimization (103, 109). Perfusion or stirred-tank bioreactors allow precise control of nutrient delivery, waste removal, and gas exchange, enabling the large-scale production of clinically relevant T-cell doses (110, 111). For example, G-Rex^®^ devices and WAVE™ bioreactors have been employed to expand TILs or CAR-T cells, maintaining high viability and functional cytotoxicity while supporting consistent expansion over multiple weeks (110). However, the adoption of bioreactor technology represents a significant capital investment and requires substantial process development to optimize parameters like seeding density, perfusion rates, and agitation. Transferring a process from a static system to a dynamic bioreactor is a major manufacturing scale-up step that necessitates extensive validation to ensure product comparability and consistency (17, 19, 102). Furthermore, maintaining sterility within these systems which involve processes that are not fully closed can be a challenge that requires robust protocols and operator training.

By carefully tuning these microenvironmental factors, including oxygen tension, nutrient availability, and mechanical cues, ex vivo culture systems can produce immune cell populations that are not only numerically sufficient but also functionally superior, with enhanced memory features, metabolic fitness, and resistance to exhaustion. However, these interventions also introduce additional process controls that must be validated and monitored under GMP conditions to be aligned with regulatory requirements (17, 19). Parameters such as oxygen concentration, nutrient supply, agitation rates, and culture duration may become critical process parameters because they directly influence cell phenotype and functional attributes (103, 110). Transitioning from static laboratory culture systems to automated bioreactor platforms frequently requires comparability assessments to demonstrate that product identity, potency, and safety remain unchanged after scale-up (19). As a result, microenvironment optimization should be developed alongside manufacturing strategies to ensure both biological performance and regulatory compliance throughout clinical development (19).

## 3 Regulatory considerations

While the previous sections highlighted technology-specific regulatory considerations associated with individual immunomodulatory strategies, the following section focuses on the broader regulatory expectations that govern ACT development, including product classification, source material qualification, manufacturing control, and clinical-scale translation.

### 3.1 Adoptive cell therapy modalities

Regulatory requirements depend on whether the therapy involves activation and expansion of immune cells ex vivo or includes genetic modification. Genetically modified products raise additional concerns such as insertional mutagenesis, off-target genome editing effects, and prolonged persistence, which necessitate more extensive preclinical testing and long-term clinical follow-up (23).

For example, TIL and CAR-T are used as representative examples of non-genetically and genetically modified ACT products respectively. CAR-T cells include a genetic modification step that is not present in TIL manufacturing, where cells are isolated from resected tumor tissue and expanded ex vivo without genetic modification (84). Due to the genetic modification of CAR-T cells there are additional chemistry, manufacturing, and control (CMC) requirements to account for the genetic modification system (23, 24, 102). **Table 3** below highlights the differences as well as examples of the approved products:

**Table 3:** Regulatory differences between genetically and non-genetically modified ACT (23, 24, 84, 112–118).

Regulatory consideration	TIL (Activation/Expansion)	CAR-T (Genetically modified)
Primary Manipulation	Tumor-derived lymphocyte isolation, activation and expansion	Receptor engineering of CAR Construct
Regulatory classification	Somatic Cell Therapy Medicinal Product (sCTMP)* Cellular and Gene Therapy (CGT) products^#^	Gene Therapy Medicinal Products (GTMPs)* Cellular and Gene Therapy (CGT) products^#^
Gene therapy designation	No	Yes
Insertional mutagenesis assessment	Not Applicable	Required
Off-target genome editing	Not Applicable	Required if genome editing was used
Vector Characterization	Not Applicable	Required (sequence, copy number, integrity)
Replication-competent virus testing	Not Applicable	Required for viral vectors
Genetic stability testing of cells from patient	Not required	Required
Mandatory Long-Term Follow up	No requirement	15 years (for integrated transgene)
Potency assay focus	Cytotoxic activity, phenotype consistency	CAR expression linked to functional activity
Approved products	Lifileucel (Amtagvi®)^#^	Tisagenlecleucel (Kymriah®)^#,*^ Axicabtagene ciloleucel (Yescarta®)^#,*^ Brexucabtagene autoleucel (Tecartus®)^#,*^ Lisocabtagene maraleucel (Breyanzi®)^#,*^ Obecabtagene autoleucel (Aucatzyl®)^#,*^ Idecabtagene vicleucel (Abecma®)^#,*^ Ciltacabtagene autoleucel (Carvykti®)^#,*^

*European Medicines Agency (EMA).

^#^U.S. Food and Drug Administration (FDA).

Several ACT products have received regulatory approval in major jurisdictions. Examples include the CAR-T cell therapies tisagenlecleucel (Kymriah^®^) and axicabtagene ciloleucel (Yescarta^®^), both of which have been approved by the FDA and EMA (112). The TIL therapy lifileucel (Amtagvi^®^) has also received FDA approval (84). While the overarching regulatory principles governing ACT products are broadly similar across major jurisdictions, important differences exist in regulatory classification and oversight. In the United States (US), the Food and Drug Administration (FDA) regulates cellular and gene therapy products through a risk-based framework that emphasizes product characterization, manufacturing controls, comparability following manufacturing changes, and long-term safety monitoring for genetically modified products (19, 24, 119). In the European Union (EU), ACT products are regulated under the Advanced Therapy Medicinal Product (ATMP) framework. Under the EU framework genetically modified products are generally classified as Gene Therapy Medicinal Products (GTMPs) and non-genetically modified cellular therapies may be classified as Somatic Cell Therapy Medicinal Products (sCTMPs) (23). Although differences exist in terminology, classification, and regulatory pathways, both regulatory agencies place strong emphasis on demonstrating product quality, consistency, safety, and efficacy throughout development, as well as maintaining product comparability when manufacturing processes are modified or scaled up (19, 23, 24, 119). Hence, developers should consider regulatory requirements across multiple jurisdictions early in process development to facilitate global clinical translation and commercialization.

### 3.2 Source material considerations

Source material control is closely linked to the choice of immunomodulatory expansion strategy, particularly when the process uses donor-derived cells, feeder cells, serum, viral vector production systems, or other human- or animal-derived materials. These materials can directly affect the safety, consistency, and efficacy of the final ACT product.

#### 3.2.1 Allogeneic vs autologous

Autologous cell therapies are derived from a patient’s own cells, while allogeneic therapies use cells from a donor. Because donor-derived products carry a higher risk of transmitting infectious diseases, allogeneic cell therapies require more stringent donor screening and testing.

According to 21 CFR §1271.75, donor screening must appropriately reduce the risk of transmitting relevant communicable diseases (120). This includes reviewing the donor’s medical records for risk factors and clinical evidence of communicable disease agents. For allogeneic donor material, manufacturers must also determine donor eligibility based on the results of screening and testing in accordance with 21 CFR §1271.75 (Screening requirements), §1271.80 (Testing Requirements), and §1271.85 (Tissue-specific testing requirements).

#### 3.2.2 Use of human/animal derived materials

Human- or animal-derived materials may be used directly in adoptive cell therapy production or indirectly in the manufacture of ancillary materials such as cell banks, viral stocks, and cytokines. Their use increases the risk of infectious disease transmission, as adventitious agents or impurities from these materials may enter the product during manufacturing. Human cells therefore require donor screening for transmissible agents, while animal-derived components such as feeder cells or serum require justification of safety and viral inactivation steps (71, 102).

Aside from these risks, there is also variability in how suppliers produce human- or animal-derived materials. Materials from different donors may vary in purity, strength, and overall quality (102). In addition, differences in starting material, pool size, and manufacturing methods mean that changing suppliers can significantly affect the safety and quality of the final ACT product.

Major regulatory agencies, including the FDA (US), EMA (EU), and PMDA (Japan), share common expectations regarding the control of human- and animal-derived materials. However, differences exist in regional regulatory frameworks and ancillary material requirements (72). For example, bovine serum is subject to different documentation, traceability, and qualification requirements across jurisdictions (72). It is therefore important to consider regulatory expectations of the intended jurisdictions when selecting human- or animal-derived materials for clinical development and manufacturing.

### 3.3 Manufacturing process

As immunomodulatory expansion strategies influence cell phenotype, proliferation kinetics, potency, and safety, the manufacturing process must be designed to control both biological variability and process-related risks. Key considerations include the use of open or closed systems, qualification of ancillary materials, and the ability to scale the process while maintaining product comparability.

#### 3.3.1 Open vs closed systems

Open, manual operations such as working in biological safety cabinets or open product transfers carry a higher risk of contamination (19). Examples of traditional open manufacturing workflows include cell activation in culture vessels, transduction in bags or flasks, and expansion using systems such as G-Rex^®^ flasks (121). As a result, they require stricter cleanroom classifications, more intensive environmental monitoring, and stronger operator qualification and gowning controls. In contrast, closed systems reduce open transfers and are viewed more favorably by regulators because they lower contamination risk and operator-dependent variability. Examples of closed and semi-automated manufacturing platforms currently used in ACT manufacturing include the CliniMACS Prodigy^®^ system, which integrates cell selection, activation, transduction, and expansion within a closed workflow (122), and the Cocoon^®^ Platform, which enables automated and functionally closed cell therapy manufacturing at the point of care (123). Hybrid manufacturing models are also common, where individual closed systems are interconnected through validated transfer procedures to create semi-automated manufacturing workflows (101, 124). However, these systems still require full validation and appropriate challenge testing (19, 101).

The use of open or closed systems would also determine the facility requirements (ISO class 5-8) to manufacture the ACT (125). With a fully closed system the requirement of the environment can be reduced, this would enable the decentralized production of ACT, bringing the manufacturing of the product closer to point of care (125). Additionally, with the use of closed systems during manufacturing, a semi-automated or automated process can be developed by combining equipment from different suppliers to cover the entire manufacturing process (18, 124).

#### 3.3.2 Choice of ancillary material

Ancillary materials are substances used during the manufacturing of adoptive cell therapies that are not intended to be part of the final product itself (72). In the context of immunomodulation strategies adopted for ex vivo expansion some examples of ancillary materials include antibody-coated magnetic beads for cell activation, and culture media supplements used to support cell activation, proliferation, or survival during manufacturing.

Because these materials directly affect both the ex vivo expansion process and the quality of the final ACT product, it is important to consider the regulatory requirements governing their use. Published standards such as USP <1043> and the more recent ISO 20399 International Standard recommend a risk-based approach for qualifying ancillary material (73, 74). USP <1043> provides a risk-categorization framework (**Table 4**), while ISO 20399 defines the responsibilities of each party involved in the use of ancillary materials during ACT production.

**Table 4:** USP <1043> risk categorization framework.

USP <1043> risk categorization framework (73)	
Tier 1 Low-Risk, Fully Regulated Therapeutic-Use Materials	Materials that are regulated as medicinal products, biologics, medical devices, or implantable materials. These materials have undergone comprehensive regulatory evaluation, including demonstrated safety, purity, identity, and performance, and therefore present the lowest ancillary-material risk. E.g. Injectable cytokines for use in cell culture media
Tier 2 Low-Risk, GMP-Manufactured Ancillary Materials with Defined Intended Use	Materials that are explicitly manufactured for use as ancillary materials and produced under an appropriate Good Manufacturing Practice (GMP)–compliant quality system. Their quality attributes, manufacturing controls, and release criteria are well characterized. E.g. Human AB serum
Tier 3: Moderate-Risk Non-Ancillary Materials Manufactured for Research or Diagnostic Use	Materials not intended for use as ancillary materials, frequently produced for research-grade, reagent-grade, or *in vitro* diagnostic applications. These materials may possess some characterization but typically lack GMP oversight or full quality-system controls. E.g. Research Use only cell culture media
Tier 4: High-Risk Materials Materials Without a Recognized Quality Management Framework	Materials lacking production under a recognized quality management system and not designated or controlled for use as ancillary materials. These materials exhibit the greatest uncertainty in terms of quality, consistency, and potential impact on cellular products. E.g. Non-qualified feeder cell lines produced in-house

It is the responsibility of the ancillary material user to qualify the ancillary material for its intended use. Qualification is the process of acquiring and evaluating data to establish the source, identity, purity, safety, and overall suitability of a specific ancillary material used in the manufacturing process (74).

The development of technologies for adoptive cell therapy manufacturing (e.g. feeder cell lines, serum replacements, non-viral gene delivery systems, or bioreactors for ex vivo expansion) requires a clear plan to transition toward Tier 1 or Tier 2 materials as programs advance to late-phase clinical trials and commercialization. While it is possible to use tier 3 materials during the early stages of clinical trials with justified risk assessment and mitigation plans, failure to transition to qualified materials would lead to clinical trial delays and costly process redevelopment at later stages.

#### 3.3.3 Scale-up for clinical trials

As clinical trials progress from early- to late-phase studies, regulatory expectations become increasingly stringent. At the same time, manufacturing processes must scale up from an academic or early-development setting to support the larger dose requirements and increased patient numbers typical of late-phase trials (17).

The scalability of the selected immunomodulatory strategy should therefore be considered early during process development, as each strategy introduces distinct manufacturing risks and control requirements. Gene-editing and gene-transfer approaches require scalable delivery methods that consistently achieve the required modification efficiency while maintaining cell viability and meeting vector- or editing-related specifications (23, 24). Cell-extrinsic strategies present different considerations: cytokines and metabolic modulators require qualified and consistent reagent supplies (72–74); feeder cell systems require control of lot-to-lot variability, contamination, and residual feeder cells (71); and non-cell-based activation systems may require validated removal of process-related materials (73, 74, 100). Similarly, bioreactor-based or controlled-microenvironment cultures require reproducible control of oxygen tension, nutrient supply, agitation, and culture duration (103, 110).

Accordingly, regulatory agencies expect sponsors to define critical quality attributes (CQAs) and critical process parameters (CPPs) early, particularly those affected by immunomodulation strategies such as cytokines, activation beads, feeder cells, or genetic modification. These attributes should guide risk assessments and comparability when moving from small-scale manual processes to larger, closed, or automated systems (19). Because changes in scale can alter cell phenotype and functional characteristics, comparability studies must be proportional to the extent of the change and the stage of development (17, 19).

Late-stage modifications often require more extensive analytical testing and may also require clinical bridging studies when the modification to manufacturing changes such that the comparability of the later-phase CGT product to the product used in early-phase clinical trial is uncertain. As processes mature, qualification of immunomodulatory reagents, validation of mechanism-based potency assays, and updates to process performance qualification (PPQ) and continued process verification (CPV) plans remain essential.

Manufacturing capacity constraints are also common in CGT, particularly during early development, and may limit the feasible cohort size (126). The ability to produce sufficient autologous batches or to consistently manufacture large allogeneic lots, combined with the size of the target patient population, can restrict enrollment (127).

During process changes intended to improve manufacturing efficiency, the product quality can be affected. If the modification results in an improvement in the quality of the product during the comparability studies, it would imply that the pre- and post-change products are not comparable. This would require additional nonclinical or even clinical studies to demonstrate the safety of the product and exclude adverse impact on product effectiveness (19).

Given these constraints, significant manufacturing changes are best introduced before initiating clinical studies intended to support a Biologics License Application (BLA) for commercialization. Late-stage changes carry a higher risk of delays and additional qualification requirements. Aligning the manufacturing scale-up changes with clinical milestones supports smoother development. By beginning clinical studies with a process that has inherent scalability, it helps prevent future disruptions as production needs grow. Regulatory agencies encourage early dialogue to align on manufacturing scale-up strategies before late-phase clinical development (128). Planning ahead ensures that immunomodulation strategies, product release criteria, and potency assays remain consistent and scientifically justified throughout development.

In addition to scalable process design, as ACT manufacturing becomes more complex and data-rich, digital tools may also support process monitoring, deviation detection, and manufacturing oversight during scale-up. Artificial intelligence (AI) enabled tools are emerging as supportive technologies for manufacturing oversight rather than as drivers of biological decision-making. FDA’s 2025 draft guidance proposes a risk-based credibility assessment framework for AI models used to generate information or data intended to support regulatory decision-making regarding product safety, effectiveness, or quality; provided that final determinations remain grounded in validated biological assays (129). Examples include a digital twin framework for the manufacturing of CAR-T cells (130, 131); Prediction of response to personalized cell therapy treatment (132).

## 4 Conclusion

Ex vivo expansion remains a central manufacturing challenge for adoptive cell therapies as the clinical trials progress towards later phases and larger doses are required to be manufactured for larger patient populations. We outlined the current landscape of these immunomodulatory strategies (cell-intrinsic & cell-extrinsic) and how they improve cell yield during manufacturing. The choice of these strategies introduces important regulatory considerations that must be addressed early in ACT development, with cell-intrinsic and cell-extrinsic approaches differing in scalability and regulatory complexity.

Immunomodulation strategies that rely on poorly characterized materials, open processes, or non-scalable platforms may be acceptable in early research settings but become limiting as programs transition toward late-phase clinical trials and commercialization. Designing expansion strategies with scalability, closed-system compatibility, and defined quality attributes in mind can reduce development risk and accelerate translation. To support successful commercialization, immunomodulatory approaches must therefore be both scalable and designed with regulatory requirements in mind. Their implementation may be further aided by emerging technologies that enhance manufacturing oversight and process control.

By aligning innovative immunomodulatory strategies with manufacturability, regulatory requirements, and complementary digital tools, the field is well positioned to translate emerging ACT technologies into widely accessible and effective therapies.
